# Facilitating implementation of the Decision-Making Capacity Assessment (DMCA) Model: senior leadership perspectives on the use of the National Implementation Research Network (NIRN) Model and frameworks

**DOI:** 10.1186/s13104-018-3714-x

**Published:** 2018-08-23

**Authors:** Suzette Brémault-Phillips, Ashley Pike, Lesley Charles, Mary Roduta-Roberts, Aruna Mitra, Steven Friesen, Lynne Moulton, Jasneet Parmar

**Affiliations:** 1 Faculty of Rehabilitation Medicine, University of Alberta, Corbett Hall, 8205 14 St., Edmonton, AB T6G 2G4 Canada; 2 Division of Elderly Care, Department of Family Medicine, Glenrose Rehabilitation Hospital, 10230 111 Ave, Edmonton, AB T5G 0B7 Canada; 3 Central West Local Health Integration Network, 199 County Court Blvd., Brampton, ON L6W 4P3 Canada; 4 Bethany Care Society, 101 17 ST, Calgary, AB T2N 2E5 Canada; 5 Edmonton Zone Continuing Care-Facility Living, Alberta Health Services, 406-10216 124 St, Edmonton, AB T5N 4A3 Canada

**Keywords:** Capacity, Competency, Decision-making, Capacity assessment, Capacity assessment model, Implementation, Sustainability, NIRN

## Abstract

**Abstract:**

Dementia and other chronic conditions can compromise a person’s ability to make independent personal and financial decisions. In the wake of an ageing population and rising incidence of chronic conditions, the number of persons who may require Decision-Making Capacity Assessments (DMCAs) is likely to increase. Legislation (e.g., Trusteeship, Guardianship, Medical Assistance in Dying) also necessitates that DMCAs adhere to legislative requirements and principles. An intentional, explicit and systematic means of implementing standardized DMCA best-practices is advisable. This single exploratory case-study examined the perspectives of senior leaders and clinical experts regarding the utility of using the National Implementation Research Network (NIRN) Model to facilitate implementation, spread and sustainability of a DMCA Model. Participants learned about the NIRN Model and discussed its application during working and focus groups, all of which were audio-recorded, transcribed, and analyzed using thematic analysis.

**Results:**

Participants found that the NIRN Model aligned well with the DMCA Model, and offered utility to support implementation, spread and sustainability of DMCA best-practices. Participants also noted barriers related to its language, inability to capture personal change, resource requirements, and complexity. It was recommended that a NIRN-informed DMCA-specific implementation framework and toolkit be developed and NIRN-champions be available to guide implementation.

**Electronic supplementary material:**

The online version of this article (10.1186/s13104-018-3714-x) contains supplementary material, which is available to authorized users.

## Introduction

Adults are presumed to be independent decision-makers regarding their personal (e.g., health, housing, associations, legal) and financial affairs. When a person’s decision-making capacity (DMC) in specific domains, however, comes into question due to diseases such as dementia and other chronic conditions, standardized Decision-Making Capacity Assessment (DMCA) processes aligned with legislation are needed. In the wake of an ageing population, increasing incidence of chronic conditions and legislative requirements (e.g., Guardianship and Trusteeship, Medical Assistance in Dying), DMCA best-practices and processes need to be standardized and better-integrated into routine care. Adherence to such processes best-positions healthcare professionals to determine person-centred outcomes that are least restrictive and intrusive, and that maximize autonomy.

The DMCA Model [1, 2] is an innovative learning and development model created in 2006 by an acute care interprofessional (IP) team. The DMCA Model aims to enable independent practitioners, IP teams, organizations, and large-scale systems to effectively conduct DMCAs. The Model outlines a standardized process aligned with provincial legislation. Its aim is to support screening and pre-assessment, facilitate problem-solving, support documentation, facilitate education and mentoring, and enable widespread implementation, spread and sustainability of DMCA best-practices.

Despite attempts to implement the DMCA Model across the continuum of care and service sectors, successful uptake, spread and sustainability of the Model has had varying results. Use of intentional, systematic, “active and planned efforts to mainstream innovation” [3, p. 582] may result in more effective DMCA Model implementation. While various implementation frameworks might be utilized [4–9], the DMCA Model most closely aligns with the National Implementation Research Network (NIRN) Model [5, 10–12] and its five overarching Active Implementation Frameworks (AIFs) [10, 12–17]. The purpose of this study was to explore the perspectives of senior leaders and clinical experts regarding the applicability of using the NIRN Model and AIFs to implement the DMCA Model in healthcare organizations.

## Main text

### Methodology

#### Design

A single exploratory case-study design was employed to document participant perspectives regarding the utility of applying the NIRN Model and AIFs to the DMCA Model. As the study was exploratory, small-scale data collection was found to be appropriate before determining specific research questions and hypotheses [18].

#### Participants

Senior leaders and clinical experts (i.e., managers, senior leaders, physicians, social workers, occupational therapists, nurse practitioners, professional practice leads) from health-related organizations across Alberta with expertise conducting DMCAs were invited to participate in a NIRN Working Group (a committee formed to examine a specific question and provide recommendations) (WGs), a NIRN Bootcamp (an intensive 2-day training workshop designed to introduce participants to use of the NIRN Model and its tools), and a focus group (FG). The in-person NIRN Bootcamp was held October 18–19, 2016. A 1-h teleconferenced FG was conducted on October 24th, 2016. Participants represented organizations at different stages of DMCA Model implementation and with varying amounts of DMCA experience. (See Additional file 1: Table S1 for participant numbers & activities).

#### Data collection

Working Groups, which included eight, 1-h biweekly teleconferences held between June 20th and September 26th, 2016, aimed to introduce and review NIRN tools/processes and AIFs and consider their utility in facilitating implementation, spread and sustainability of DMCA processes. In advance of the WGs, participants reviewed selected resources on NIRN’s Active Implementation (AI) Hub [17].

#### Data analysis

Working Groups and the FG were audio-recorded, transcribed, and entered into NVivo 11. Thematic analysis, which was conducted by research assistants following methodology outlined by Braun and Clarke [19], employed both an inductive and deductive approach.

### Results

Participants identified facilitators, barriers and recommendations regarding the use of NIRN tools/processes with the DMCA Model. These are described and tabulated in the following section. (See Tables 1, 2, 3 for themes and related quotes).

**Table 1:** Facilitators of use of the NIRN Model

Category/theme	Supporting quotes
The NIRN Model is effective and gives credibility to implementation of best-practices	*“The NIRN Model has a strong practical element… it’s a very powerful framework.”* (2016-10-24) *“The utility is fantastic. What I found of value is just knowing that it is there.”* (*2016-10-24*) *“It’s a structure to be followed to ensure practice implementation, things are done appropriately. [We] have been talking about that in the past three years; we felt we weren’t being taken seriously. Now all of the sudden we are.”* (*2016-09-12*) *“[NIRN gave the language, credibility to speak about and frame implementation]. We all know in a practical sense that it is going to work. When you have a framework, people take things more seriously. Administrators in the group appreciated and understood that there was some research around the NIRN Model and that there was an actual tool we were following that could capture where the holes were. It really supports the work.”* (*2016-09-12*) *“It facilitates adoption and practice change, and if you do it well, fidelity to the practice.”* (*2016-10-24*)
The NIRN Model, AIFs and DMCA processes align well	*“[The NIRN Model] offers something familiar, and aligns with and makes explicit what people already do.”* (*2016-10-24*) *“When I look at the DCMA Model and what the NIRN components suggest, both align very well from what I am seeing so far. Haven’t seen anything in the NIRN that hasn’t been introduced or utilized as people have been implementing decision-making capacity assessment processes.”* (*2016-10-24*) *“I think the NIRN Model has a lot of use for the DMCA pre*-*assessment because it is such a complex process that we are using. To have something as detailed and that allows you to explore all the different components of it made a lot of sense in my mind… I think it fits really well with this go around trying to implement this DMCA pre*-*assessment process.”* (*2016-10-24*) *“It’s a good match, but only if you have someone guiding the process.”* (*2016-10-24*)
The NIRN Model provides a clear process for implementing DMCAs	*“The NIRN Model facilitates a more global approach to implementing something so that you have a greater chance of it being permanently adopted and having that sustained change.”* (*2016-10-24*) *“The NIRN Model has an enormous amount of detail to it, but it does provide excellent framework for figuring out where to go next.”* (*2016-09-26*) *“Reflecting on items in the NIRN Model allowed me to distill where things are going well and where things aren’t and look at foundational things that may have been overlooked otherwise… It helped guide how to present things, influence corporate office around policy, support all these different sites, determine who needs to be involved, how to disseminate information and go from there."* (*2016-09-12*) *"There is a lot of content, but as you are working your way through, it cues you to think about aspects that you might not have in place, to consider where are we… I like how it keeps re*-*cueing you to think, “did you look at that”, “do you have a plan written out”, “how are you going to coach that”, “did the plan go out”, [I] see some great value there.”* (*2016-08-15*) *“It allowed me to see how the organization I worked for had implemented things. [If you] had not gone through a process like this and then you wonder why certain initiatives don’t work and others do… it gives a very structured path and allows you to go back into it and double check: has anything been missed, are there areas that needs to be revisited and those are the things I really liked about it.”* (*2016-09-26*) *“For the process itself, I think the way it is set out and the way it is displayed*—*how it follows the steps, it’s just set up really clearly and I think that is what I wanted to see. There is a structure to it and there is a way to follow through.”* (*2016-09-26*) *“It makes things explicit and everyone is on the same page.”* (*2016-06-20*)
Usable innovation: the NIRN Model and AIFs challenge professionals to define DMCA best-practices	*“We are currently… trying to gain consistency with our terminology… I think all of us working through the process also helps confirm that at this level, we all have similar sort of ideas about what should be and what we think should be happening. I think our gold standard of what the practice is would be identified as quite similar. [The challenge is to] come up with something that is concise and using terminology that is consistent.”* (*2016-09-12*) *“The Practice Profile Activity really helped to describe the gold standard of DMC processes, what was acceptable and unacceptable.”* (*2016-10-24*) *“Fidelity Assessments support determination of whether decision*-*making capacity assessments are being done well.”* (*2016*-*09*-*26*) *“Fidelity indicators help to really look at what will indicate what is actually happening.”* (*2016-09-26*)
Stages of implementation and the analysis tool enables systematic evaluation of activities and the development of an action plan to improve the success of ongoing implementation efforts	*“It looks very comprehensive.”* (*2016-08-15*) *“A lot of information in terms of utility. It systematically encourages one to look at the different stages… useful.”* (*2016-08-15*) *“[The tool] systematically encourages one to look at the different stages… useful to think about where we are with the DMCA…gives you a sense of where you are at in the process which is useful because sometimes these things feels very complex. [Stages of Implementation Analysis] can be used as a tool to keep one on track*—*especially in initial implementation, when it is a new thing and it keeps everyone on the same page.”* (*2016-08-15*) *“I like how it is broken down with the different levels*—*exploration, installation, initial implementation, full implementation.”* (*2016-08-15*) *“The advantage of using a system like this*—*you can be at different stages even within the stages, different places: i.e. initiating things, thinking ahead and planning.”* (*2016-08-15*) *“When we look at how we implement [the DMCA process]…, we did a number of processes without following a specific model which I think was good. But what this does is that it keeps you more accountable. We have some practices and processes that weren’t as effective*—*could have revisited and been more objective: how can we strengthen this, what do we need to do differently, what can we do next? [We] could use a process like this to go back and revisit that.”* (*2016-08-15*) *“I like [the headings]: in place, not in place, partially in place, seems like clear questions… if you went through it, you would come up with some specific conclusion, build on your plan, and specific action items that would need to be put in place that might be overlooked otherwise to hinder successful adoption.”* *“When looking at difficulties*—*why is the implementation not working, why is it not sustainable, where are the things breaking down, how do we make it sustainable, I realize in looking at the tool*—*it’s about the detail of it that is significant*—*many of the things we can put that is not so specific to the site I do feel that in getting into such detail, it might be the key to determine what is working and what is not.”* (*2016-08-15*)
Exploration: enables thorough assessment of site readiness prior to implementation	*“I like how the hexagon tool pieces together all the things we need to look at and consider before we actually truly implement…it helps to sets out what exactly it is that we will need to figured out, talk about, can use to actually engage the SW, OT, other stakeholders in it in terms of the need, fit, evidence… bringing it more down to the tangible what we can do.”* (*2016-08-15*)
Installation: ensures that appropriate components (e.g., education, organizational structures, buy-in, champions, implementation team, a communication plan) are in place so as to facilitate successful implementation	*“Having people who are engaged and passionate about the topic who are willing to take on the load [made implementing the practice possible].”* (*2016-11-25*) *“We are going to implement the policy anyways, so it will be in place.”* (*2016-11-25*) *“I think the most important thing that has happened is education…” “The training went well. The implementation of the training afterwards not so much, because we are not doing this everyday so it is going to be a process and take longer to get everyone up to speed.”* (*2016-11-25*) *“Having meetings scheduled made implementation possible”.* (*2016-11-25*) *“Buy in from managers and administrators.”* (*2016-11-25*)
Implementation of best-practices is a process	*“I’m just happy that were actually putting best*-*practices into place.”* (*2016-11-25*)
Making the process explicit is helpful	*“It is interesting to see at an organization level how it has been implemented, so how that background work gets done as well and how person*-*centred care has been at the forefront. That’s really what the DMCA model has been, so it’s been really good to see how that has unfolded over the last couple of months.”* (*2016-11-25*)
Learning strategies (e.g., use of huddles and worksheets) are supportive of implementating the DMCA best-practice	*“We attached huddles to the IP rounds because we didn’t want to add another meeting.”* (*2016-11-25*) *“If you didn’t have the huddles… it would’ve been effective. You needed the huddle to get a picture of what the individual was. It helped with educating.”The huddles made people see that there was something… happening. And for the problem solving piece too.”* (*2016-11-25*) *“The huddles really help work through problems. Worksheets are a nice guide for huddles, really helps drive our huddles”.* (*2016-11-25*) *“I really liked the worksheet. It really helped guide and outline the process and as far as education goes, I think it is one of those things that you do enough of it then you learn how to do it.”* (*2016-11-25*)
Mentorship and consultants are essential during implementation	*“We always had someone to go to or a resource when they were stumped.”* (*2016-11-25*)
Implementation teams are critical to ensuring all aspects of implementation	*“When I looked at our initial group as we embark on this project, we were all involved*—*a lot of us are front line staff who want to make a moral and ethical change. We looked at educating our peers and being an example and model, resource to them. But we didn’t have authority to look at things like performance*—*providing feedback to upper level of the organization, to have other key changes take place….Things are able to be picked up within various part of the organization from front line staff and some things are reserved for senior leaders.”* (*2016-08-15*)
Implementation drivers facilitate operationalization of the best-practice	*“The drivers piece is very important and a good reminder for us of all the various pieces that have to be in place in order to get this to work.”* (*2016-08-02*) *“The thing I like about the NIRN is identifying different drivers and the processes together, we came up with a number of concrete action plans that we can do to move forward. So there is an overall feeling of positivity that we are identifying goal along the way that we are achieving but also realize that there are very significant issues that need to tackle as they are starting to come up…”* (*2016-09-26*)
Leadership drivers	*“Leadership at all levels but also in parallel lines.”* (*2016-08-02*) *“Site leaders that are actually engaged and attached to the mentoring team.*” (*2016-11-25*) *“[Having] the right people… at the table almost starting into the initial stages of implementation…getting the people at the table that is actually part of what we have in our drivers. We acted on those right away and brought those people to the table. It changes the dynamic and makes the process richer.”* (*2016-09-26*)
Competency drivers	*“Is it necessary to have a champion? I would say unequivocally yes. Would it be helpful to teach others what it means? Yes, if we were trying to learn from scratch, it would be the blind leading the blind… Experiential knowledge*—*it was key she had been through it with other groups before so she can give you that soft knowledge.”* (*2016-10-24*) *“For some of these changes that take place, there is a time component where people have to set time aside to do training and to have the system support.”* (*2016*-*08*-*02*) *“Coaching and training plan were really good. They make it very overt who is going to do what*—*very clear.”* (*2016-09-26*)
Organizational drivers	*“Time… to sit down with people who are clinician and those who have the senior leader’s eye to facilitate practice change.”* (*2016-09-12*) *“Dedicated human resources makes implementation possible. For example, one facility has a .8 FTE for one year to focus on capacity assessment implementation and sustainability.”* (*2016-10-24*) *Documentation: “The structured note: it is key to success. It’s a collective space where people can go and input data, make it more real, as opposed to places where there is no system in place.”* (*2016-08-02*)
Improvement cycles support adaptation of processes to ensure success	*“You have to go back part way and then move forward*—*so we have been engaging in that cycle… carry forward what you have already, but add pieces that you need.”* (*2016-09-26*)
Evaluation: the NIRN framework provides measurable components and helps service providers identify organizational gaps and barriers to the uptake of the best-practice	*“What I like is that it’s broken down into measurable components.”* (*2016-06-20*) *“I like how it’s broken down with the different levels*—*exploration, installation… It cues you as you work through to think about aspects that you might not have/have put in place… consider where are we.”* (*2016-08-15*) *“It’s helpful that there’s a framework to use to ask: who, what, where, when, how, why. When applied to CHOICE day program, seniors clinic, long*-*term care, that is where we found that as we were working through [the NIRN framework], we found the gaps quite clearly…as a result, they helped guide how we present and influence our umbrella, organization, our corporate office around policy, how to support all these different sites, who needs to be involved, how to disseminate information and go from there.”* (*2016-09-12*) *“Probably having tick marks as a starting point is a good start… Percentage is difficult evaluate*—*what does 60 versus 80% mean?”* (*2016-08-15*) *“One of the nice things of a tracking tool is that it keeps the momentum going.”* (*2016-08-15*)
Communication: NIRN helps to facilitate communication during implementation	*“Adds clarity regarding the individual responsible and who is the next step to assign accountability.”* (*2016-08-15*)

**Table 2:** Barriers to the use of the NIRN Model

Category/theme	Supporting quotes
Gaining buy-in from various levels and disciplines can be challenging	*“Trying to get other people to buy in would be hard.” (2016*-*08*-*15); [Adopting it] would need a lot of buy in and a lot of work (2016*-*10*-*24); “There was not a lot of physician buy in. Having the medical director involved too was essential.” (2016*-*11*-*25); “Some projects in the past have failed because there was no buy in from the leadership… You do need a driver*—*someone that will be in charge.” (2016*-*08*-*02); “If the leadership is not behind you, a lot of time things fall down. So you need them on board for things to move. No matter what the level of motivation is at the grass roots, without that higher level support*—*things tend to die because there are competing demands and people don’t have time to do everything. To have the time to do it and focus*—*make priority, then leadership has to be on board.”* (*2016-08-02*)
There is a tendency to not clearly articulate outcome measures when introducing best-practices	*“From the point of view of education or practice change, we often think about what we want the outcome to be, but we don’t often articulate it in a very clear way and then are incredibly surprised when we don’t get what we expected.” (2016*-*06*-*20)*
NIRN lacks outcome measures and does not capture change at the person level	*“It does not address the individual change process. There are certainly some principles in the NIRN material on how to involves people, and that you should involve them in order for them to be invested in the change, but it pretty well ignores the whole psychological part of change as an individual” (2016*-*10*-*24); [It doesn’t, however,] “offer ways to capture outcome data” (2016*-*10*-*24);The process of implementation takes time. “We know implementation takes 3*-*5* *years.”* (*2016*-*11*-*25*)
Using the NIRN Model and AIFs is resource-intensive	*“For me, it was a little sobering to hear timelines being quoted of 2*–*4* *years when using this process from beginning to implementation that sometimes can be overwhelming to present to a team or organization when they think about resources allocation and so forth… and again, the barriers are always the buy in making sure that the right people are knowledgeable and educated about the process and that they are willing and interested to be involved in the amount of time and effort that this is going to take up front.”* (*2016*-*10*-*24*)
It is highly detailed and time-intensive to learn	*“It was a significant learning curve to adjust to the language and the different practice i.e. the different worksheets and things like that. [The tools] was useful as we went through it but it was time intensive and I think it was hard to get my head around some of the pieces that it was looking for.”* (*2016*-*10*-*24*)
The language of the NIRN frameworks is not specific to healthcare	*“Language*—*need it to be specified from education to healthcare and DMCA.”* (*2016*-*08*-*15*)
Usability is a potential concern	*“I couldn’t use these tools and figure it out by myself.” (2016*-*08*-*15) “You have to be pretty disciplined to use [the NIRN tools]. I wonder about the usefulness in a very concrete way in the real world*—*worry that there’s just too much going on there. I like the content, but I find it seems a bit overwhelming.”* (*2016*-*08*-*15*)
Organizational drivers regarding documentation can be difficult to implement on a system-wide basis	*“Part of what we continued to lack is the consistent documentation and consistent expectations of documentation… If we are all using the same documents and the judge could expect to see the same documents, as a system we would expect those same documents and we would expect everyone works with the same tools. I still think that is going to be an identified issue despite how hard people have worked to create the worksheet and the rest of the array of tools. [I’m] not sure that everyone has bought*-*into is using the ones we had.”* (*2016*-*08*-*02*)

**Table 3:** Recommendations regarding use of NIRN Model with the DMCA Model

Category/themes	Supporting quotes
Using the whole NIRN implementation process is advisable	*“Need to follow the NIRN framework and stay true to it… While the tools can be used individually, it really is a whole process.”* (*2016*-*10*-*24*)
Buy-in at and participation from various levels of leadership and disciplines is needed	*“Buy in from managers and administrators.”* (*2016*-*11*-*25*) *“Having the freedom and flexibility to plan get together.”* (*2016*-*11*-*25*) *“Getting buy in from staff is something we need to focus on.”* (*2016*-*11*-*25*) *“Time is needed to sit down with people who are clinicians and those who have the senior leader’s eye to facilitate practice change.”* (*2016*-*09*-*12*) *“You need leadership at all levels, but also in parallel lines.”* (*2016*-*08*-*02*)
Mentorship and consultants are essential resources during implementation	*“Someone to go to or a resource when they were stumped.”* (*2016*-*11*-*25*)
Implementation: access to champions/teams are critical to ensuring all aspects of implementation	*“Is it necessary to have a champion?*—*unequivocally yes*—*someone who understands it really well so that can build on it right away.”* (*2016*-*10*-*24*) *“Having an implementation team or individual to guide the process is ideal”* (*2016*-*10*-*24*) *“…The implementation team… that… guides the process had worked very well… That would be the ideal scenario, but at least one person to shoulder it and be the standard bearer as the process is being utilized.”* (*2016*-*10*-*24*) *“If there were an* in situ *implementation team that would [simplify the tools] and take that forward to the group that is in the process and make the tools like the plan, to be usable for them, to be practical. I don’t think they need to have the full 12 course meal of the NIRN, but they could have a reasonable takeout version of it so that it is practical, useable, and it’s supporting them and they can sustain it but still holds to the fidelity of the model.”* (*2016*-*10*-*24*)
Training in use of the NIRN Model is needed	*“I would be more optimistic if I had good partners to work with who have a good understanding of [NIRN tools]. If I’m just on my own, it’s overwhelming.”* (*2016*-*08*-*15*) *“Use of learning strategies to support learning of the DMCA Model (e.g., huddles and worksheets) and NIRN Model (working groups) is advisable.”* *“IIf you didn’t have the huddles… it would’ve been effective. It helped with educating.”* (*2016*-*11*-*25*) *“The huddles really help work through problems. Worksheets are a nice guide for huddles, really helps drive our huddles.”* (*2016*-*11*-*25*) *“I really liked the worksheet. It really helped guide and outline the process and as far as education goes, I think it is one of those things that you do enough of it then you learn how to do it.”* (*2016*-*11*-*25*)
DMCA-specific NIRN tools would facilitate use	*“[The NIRN] tools would be usable if we prepare them and sell them in the right way*—*may need to sit down and decide what part can be done in a bigger group and what part needs to be done in the smaller group.” (2016*-*08*-*15)* *“Simplify the tools… make things a little less jargony for certain organizations depending on who is becoming involved in the process. I think all of that would be helpful.”* (*2016*-*10*-*24*)
Sustainability efforts are needed throughout implementation and spread efforts	*“It’s important not to underestimate the need to think ahead to consider sustainability planning while in initial implementation, although you don’t get to that until the full implementation stage.”* (*2016*-*08*-*15*) *“We need site leaders that are actually engaged and attached to the mentoring team otherwise it will not sustain or implement.”* (*2016*-*11*-*25*)

#### The NIRN Model, Active Implementation Frameworks (AIFs) and Tools [5, 10–17]

Participants valued resources and tools available through the AI Hub, finding them to be accessible, powerful, and useful. They anticipated that use of the tools would positively impact adoption and practice change, clarify and give credibility to implementation processes, and improve fidelity to DMCA best-practices.

#### The DMCA Model aligns well with the NIRN Model and AIFs

Participants recognized that the DMCA Model includes an implementation strategy and capacity-building processes. They concluded that the NIRN tools would support adoption of the DMCA Model at the provider, organizational, and system levels, and ensure fidelity to DMCA best-practices.

#### The DMCA Model [1–3] is a usable innovation [20]

Participants appreciated the DMCA Model’s person-centred approach, alignment with provincial legislation, problem-solving strategies, and emphasis on determination of least restrictive and least intrusive solutions to declining DMC. Participants acknowledged the need for further discussion among service providers to better define critical components of DMCAs and gold or acceptable standards so as to ensure consistency of DMCA administration.

#### Implementation stages [5]

Participants indicated that the NIRN’s stages of implementation and accompanying analysis tool is valuable for assessing and communicating an organization’s current state of delivery of DMCA services. Participants evaluated implementation and sustainability processes pro- and retrospectively using the Analysis Tool, considering reasons that implementation, spread and sustainability of the DMCA may have been less effective and ways to mitigate barriers.

#### Implementation drivers [21, 22]

Participants discussed the applicability of implementation drivers (leadership, competency and organizational) to the DMCA Model: *Leadership drivers*—participants emphasized that senior leader buy-in/support and the availability of champions is critical to successful implementation; *Competency drivers*—clinician competencies were noted to be essential to effective DMCA practice. Knowledge experts with a dedicated role, protected time, and critical attributes (i.e., confident, knowledgeable, credible, trusted, collaborative), were seen as being best-able to advance DMCA practice. Resources and ongoing education are also critical to the sustainability of the DMCA Model; *Organizational drivers*—successful DMCA Model implementation requires that organizational drivers be put in place including intake and documentation processes, mentoring teams, education/training, and medico-legal-ethical supports.

#### Implementation teams [5]

Participants identified parallels between NIRN implementation teams and DMCA Advisory Committees and Mentoring Teams. Engagement of key players early in the implementation process was noted to maximize success.

#### Improvement and communication cycles [5, 23]

Plan-Do-Study-Act (PDSA) and policy-practice communication cycles were routinely employed by participants implementing the DMCA Model. More deliberate communication efforts would be helpful. NIRN tools may support such efforts.

#### Evaluation [5, 24]

Participants appreciated that a systematic implementation framework can make successes and potential gaps more explicit. The NIRN tools and AIFs helped participants identify what was/was not going well regarding the implementation of DMCA best-practices.

#### Barriers to use of the NIRN Model and AIFs

Participants identified barriers related to language, resources and complexity. Some participants struggled to interpret the NIRN and AIF resources (often education-specific) into the healthcare context. They suggested adapting the language to be DMCA-specific and developing a NIRN-informed Implementation Framework and Toolkit for the DMCA Model inclusive of a Practice Profile. They also commented on the time needed to learn and apply the NIRN Model and AIFs. Participants felt that partnering with a NIRN implementation specialist or establishing a NIRN interest-group would be valuable. As implementation can be lengthy and demanding, (requiring an average of 2–4 years), strong buy-in, commitment, and a clear process is needed. To increase the likelihood of success, use of the NIRN implementation process in its entirety is advisable.

#### Recommendations regarding use of the NIRN Model with the DMCA Model

Participants insisted that use of and training in the NIRN Model and AIFs is needed, coupled with senior leader buy-in and access to a NIRN champion or implementation team.

### Discussion

This paper reports on a single-exploratory case study that considered the perspectives of senior leaders and clinical experts regarding the applicability of the NIRN Model and AIFs in supporting implementation, spread and sustainability of the DMCA Model. The emerging themes suggest that a NIRN-informed DMCA-specific implementation framework and toolkit would be helpful in guiding independent healthcare professionals, IP teams, and organizations when attempting to embed DMCA processes into routine practice. Participants also identified challenges associated with use of such a framework to support DMCA Model implementation.

Consensus from the participants was that the NIRN Model, AIFs and tools were valuable and aligned well with the DMCA Model and best-practices. They indicated that implementing the DMCA Model would be better managed using such an explicit, intentional, and systematic framework. The NIRN tools helped participants identify readiness for DMCA Model adoption, implementation stages and strategies, and successes, barriers and gaps related to previous implementation attempts. Examination of the NIRN tools stimulated reflection on the importance of champions, fidelity to DMCA practices, and evaluation and sustainability of the best-practice. Participants anticipated that utilization of the NIRN AIFs would increase credibility of the implementation and the evaluation processes. Overall, the NIRN Model was found to provide a clear framework for implementing DMCAs.

Barriers were also identified. The NIRN implementation process was found to be resource-intensive and its lengthy timeline was concerning for those who felt that staff turnover may compromise the process. Some participants also indicated that it would be difficult to apply the NIRN tools and AIFs without the support of dedicated implementation specialists. Participants further noted that, while the NIRN Model facilitates evaluation at the system and process levels, it is less effective in so doing at the service provider level. Outcome measures to evaluate the effectiveness of implementation were also found to be lacking. Finally, the language is more specific to educational rather than healthcare environments.

Specific to the DMCA Model, participants highlighted commonality and variability regarding DMCA best-practices. Terminology regarding, conceptualization and the intent of the DMCA Model was a point of discussion and at times sensitivity. “Process” appeared to be a more agreeable term than “Model” when referring to DMCAs. Further, although similar concepts were used by participants regarding DMCA processes, inconsistencies appeared. For example, it was challenging to define DMCA processes, and isolate assessment components, essential information to be gathered and by whom, and gold standard indicators. Reflections on DMCA processes stimulated through consideration of the NIRN Model and tools facilitated greater dialogue, collaboration, DMCA Model development, and consistency of DMCA practice at organizational, zonal and provincial levels.

Participants recognized the utility of the NIRN Model, AIFs and tools. As the NIRN Model provides a clear process and framework for implementing DMCA best-practices, organizations might utilize it to support implementation of the DMCA Model and processes. Such use may support local as well as widespread adoption of the DMCA best-practice processes and ensure fidelity. Commitment to its use, however, would necessitate buy-in at the leadership levels and access to NIRN-specific resources.

### Conclusions

The goal of the DMCA Model is to effectively integrate DMCA best-practices into routine service provision. Study findings support the future development and evaluation of a DMCA-specific NIRN-informed implementation framework and toolkit to facilitate implementation. Decreased resource requirements would result from development of such an implementation framework and toolkit and support best-practice uptake. Access to a dedicated NIRN-champion or implementation team would further enable the uptake of DMCA and other evidence-based practices, drive change and offer leadership.

With respect to the implementation of the DMCA Model, it is recommended that organizations consider using the NIRN Model, AIFs and tools to support the uptake of DMCA processes, and ensure sustainability of and fidelity to DMCA best-practices. Employing the NIRN Model as a framework for implementation, sustainability and spread of the DMCA Model would offer an explicit, intentional, and systematic process for implementing and sustaining DMCA processes. While time and resources are required to do so, not employing an implementation model can result in failure to implement or sustain the best-practice, demoralization of staff, and loss of time and resources. Rather than being focused on costs associated with the use of an implementation framework, however, perhaps the better question is whether or not organizations can afford not to use a process and tools that can best-position teams for integrated, sustained and successful implementation of DMCA best-practices.

## Limitations

This study has a number of limitations. While a number of organizations from across the continuum of care participated in this project, the results focus on the perspectives of 13 senior leaders and clinical experts who voluntarily attended some, though not all of, the working and focus groups, and a NIRN Bootcamp; perspectives of frontline staff were not captured. As a result, reported findings are not necessarily representative of all organizations that have implemented the DMCA Model, nor are they generalizable to other organizations.

## Additional file

**Additional file 1: Table S1.** Project Activities and Participants. This table includes the number of participants that attended each of the working groups, NIRN Bootcamp and focus group. Also included is the aim and focus of each of the project activities.

## Abbreviations

AI: Active Implementation
AIF: Active Implementation Frameworks
DMC: decision making capacity
DMCA: decision making capacity assessment
FG: focus group
IP: interprofessional team
NIRN: National Implementation Research Network
PDSA: Plan-Do-Study-Act
WG: Working Group
