# Supplementary material for: Facilitating implementation of the Decision-Making Capacity Assessment (DMCA) Model: senior leadership perspectives on the use of the National Implementation Research Network (NIRN) Model and frameworks
Source: BMC Res Notes. 2018 Aug 23;11:607. doi: 10.1186/s13104-018-3714-x (PMC6107947; doi:10.1186/s13104-018-3714-x)
Supplement: Supplementary file 1 — Additional file 1: Table S1. Project Activities and Participants. This table includes the number of participants that attended each of the working groups, NIRN Bootcamp and focus group. Also included is the aim and focus of each of the project activities. [file 13104_2018_3714_MOESM1_ESM.docx]

Table S1: Project Activities and Participants

| Project Activity | # of  Participants | Aim and Focus |
| --- | --- | --- |
| Working Groups |  | **Aim:** To learn about NIRN tools/processes and AIFs, and consider their application to the implementation, spread and sustainability of DMCA processes |
| 1 (2016-06-20) | 12 | Introduction to NIRN and the AI Hub |
| 2 (2016-07-04) | 12 | Usable innovation: DMCA processes |
| 3 (2016-07-18) | 13 | Exploration: Hexagon tool to determine readiness for implementation |
| 4 (2016-08-02) | 8 | Implementation stages and drivers: Part I |
| 5 (2016-08-15) | 9 | Implementation stages and drivers: Part II |
| 6 (2016-08-29) | 9 | DMCA Practice Profile: Part I |
| 7 (2016-09-12) | 8 | DMCA Practice Profile: Part II |
| 8 (2016-09-26) | 6 | Fidelity, Improvement Cycles, Implemaps, Communication and Evaluation Plans |
| NIRN Bootcamp  2016-10-18/19 | 7 | **Aim:** To introduce the fundamentals of the NIRN approach to integrating best practices into clinical work, and facilitate application of the processes to clinical practice |
|  |  |  |
| Focus groups  2016-10-24 | 7 | **Aim:** To solicit summative feedback regarding the utility and applicability of using NIRN processes and tools to facilitate implementation, spread and sustainability of DMCA processes |
